# Fundamental Study of Decellularization Method Using Cyclic Application of High Hydrostatic Pressure

**DOI:** 10.3390/mi11111008

**Published:** 2020-11-15

**Authors:** Daiki Zemmyo, Masashi Yamamoto, Shogo Miyata

**Affiliations:** 1 Graduate School of Science and Technology, Keio University, 3-14-1 Hiyoshi, Yokohama 223-8522, Japan; nekonigohan@a6.keio.jp (D.Z.); ironmasashi3434@a3.keio.jp (M.Y.); 2 Department of Mechanical Engineering, Faculty of Science and Technology, Keio University, 3-14-1 Hiyoshi, Yokohama 223-8522, Japan

**Keywords:** decellularization, high hydrostatic pressure, cyclic hydrostatic pressure, nuclear envelope destruction, NB1RGB

## Abstract

Decellularized tissues are promising materials that mainly consist of extracellular matrices (ECMs) obtained by removing all cells from organs and tissues. High hydrostatic pressure (HHP) has been used for decellularization to remove cells physically from organs or tissues rather than by chemical methods. However, ultrahigh pressure induces denaturation of the ECM structure. In this study, we examined the effects of cyclic HHP at low and high pressures on the cell membrane structure to establish a novel decellularization method that enables decellularization without the denaturation of the ECM. A decellularization device using cyclic HHP (maximum pressure: 250 MPa, cycle number: 5) was developed. NB1RGB cell suspension was injected into a plastic bag to be subjected to cyclic HHP. After applying cyclic HHP, the amount of DNA inside the cells and the morphological changes of the cells were evaluated. As a result, the amount of DNA inside the cells decreased after the cyclic HHP compared to the static HHP. In addition, cyclic HHP was suggested to promote the destruction of the cell and nuclear membrane. In conclusion, it was revealed that the cell structure could be denatured and destroyed by cyclic HHP at a lower level than that of previous approaches.

## 1. Introduction

Tissue engineering is a discipline that aims to regenerate tissues and organs using living cells and scaffold materials. In tissue engineering, organs and tissues are composed of three elements: cells, scaffold materials, and physiologically active substances [1]. Scaffold materials act as substitutes for cell adhesion to promote cell activities. Bioabsorbable polymers are used as raw materials for scaffold materials, and these polymers are classified into two types: natural polymers and bioabsorbable synthetic polymers. Natural polymers include collagen, gelatin, and polypeptides. They are used in hydrogel form as well as porous materials [2,3]. Commonly used synthetic polymers include poly L-lactic acid (PLLA), polycaprolactone (PCL), polyglycolic acid (PGA), and poly-lactic-glycolic acid (PLGA) [4,5,6,7]. Natural polymers contain cell adhesive molecules to promote cell adhesion, proliferation, and cell differentiation; however, they lack sufficient mechanical strength [8,9,10]. In contrast, synthetic polymers grater mechanical strength, but do not interact as favorably as biologically derived scaffolds [11,12]. In recent years, decellularized tissues obtained by removing cells from tissues obtained from humans or animals have been used as scaffold materials [13,14]. Decellularized tissue has several advantages compared to other scaffold materials: it enables the utilization of the structure and composition of three-dimensional extracellular matrices (ECMs) of living tissue, and to deliver many cytokines [15]. Recently, decellularized tissue has been clinically applied as a scaffold to regenerate heart valves and blood vessels [16,17].

To manufacture decellularized tissue, cells in native tissues are removed to simultaneously suppress tissue damage [18]. Decellularization is performed by chemical or physical methods [19,20,21]. In chemical methods, surfactants, such as anionic surfactants and nonionic surfactants, and enzymatic agent such as trypsin have been used for removing cells from native tissues. Anionic surfactants (ex. sodium dodecyl sulfate (SDS)) remove cells by solubilizing cell and nucleic membranes. Non-ionic surfactants (ex. triton X-100) remove cells by disrupting DNA–protein, lipid–lipid and lipid–protein interactions [22,23]. However, there are concerns about the risks of destroying or denaturing the structures of ECMs in tissue and the toxicity of the surfactant remaining in the decellularized tissue [24].

As alternatives to chemical methods, physical methods such as freeze-thawing, nonthermal irreversible electroporation (N-TIRE), and application of high hydrostatic pressure (HHP) have been studied for decellularization. In such physical methods, there is no risk of residual reagent as in chemical methods. Freeze-thawing is a method to remove cells by repeated freezing and thawing [25,26]; however, it must be combined with a chemical treatment. N-TIRE preserves the structure of the ECM as well as blood vessels; however, this process is only applicable to a limited tissue size range [27,28].

HHP application methods to destroy cell membranes have been studied to reduce the usage of surfactants and to decellularize large tissues. This method poses no risk of surfactant remaining, as caused by other decellularization methods using chemical reagent treatment, and it can easily retain the structures of ECMs. Several studies have been reported on the decellularization of biological tissue by applying HHP. For example, ultra-HHP of 980 MPa was applied for 10 min to decellularize porcine carotid artery and rat uterine tissue. It was confirmed that the decellularized tissues functioned well as a scaffold material after transplanting in vivo [29,30]. However, ultra-HHP application poses a risk of damage to native ECM structures. The relationship between the magnitude of HHP and the denaturation of collagen was also reported. It was demonstrated that a pressure of 200 MPa did not affect the cooperativity of transition; by contrast, a pressure above 320 MPa increased its enthalpy and destabilized the collagen [31]. Therefore, it is considered that an HHP application of 320 MPa or less is suitable to retain the microstructure of ECMs to suppress collagen denaturation. Shimada et al. reported the effect of HHP on the structure of yeast cells. The pores in the nuclear membrane were observed at 100 to 200 MPa of HHP, and the nuclear membrane was destroyed by HHP over 300 MPa [32]. In addition, Kato et al. constructed a model for cell membranes using proteins and lipids derived from porcine kidneys and reported that hydrostatic pressure under 100 MPa induced a decrease in the fluidity of the lipid bilayer. At 100 to 220 MPa, the dissociation of, and/or conformational changes in, the protein subunits were observed [33]. At 220 MPa and above, the entire lipid membrane structure was irreversibly destroyed and the cell membrane was fragmented.

Therefore, the lower HHP treatment has the potential to decellularize without damaging ECM structures. Additionally, it was reported that the strength of the cell membrane decreases with cyclic mechanical deformation because the strength of materials decreases when mechanical stress is applied repeatedly [34]. In this study, we hypothesized that the strength of the cell membrane would be weakened by repeatedly applying lower and higher hydrostatic pressure, and also hypothesized that cyclic application of HHP could promote the inflow and outflow of intracellular fluid through the cell membrane to induce its destruction. The purpose of this study is to establish a novel decellularization methodology using cyclic HHP treatment from lower to higher pressure. Furthermore, to validate the utilization of cyclic application of HHP for decellularization, a cell suspension was subjected to cyclic HHP to evaluate the effect of the cyclic HHP treatment on cell viability and microstructure.

## 2. Materials and Methods

### 2.1. NB1RGB Cell Culture

In this study, the effect of HHP on normal human skin fibroblasts (NB1RGB, RIKEN BRC, Kyoto, Japan) was evaluated to validate a physical decellularization method using HHP. NB1RGB cells were maintained in alpha-modified Eagle minimum essential medium (MEM α, Thermo Fisher Scientific Inc., Waltham, MA, USA), supplemented with 10% fetal bovine serum (FBS, Sigma Aldrich, St. Louis, MO, USA) and 1% antibiotic–antimycotic (Nacalai Tesque, Kyoto, Japan) in a humidified CO_2_ incubator (5% CO_2_ at 37 °C). From a cryopreserved stock, NB1RGB cells were passaged twice before the HHP application experiments. Harvested NB1RGB cells were suspended in a culture medium of MEM α without phenol red supplemented with 10% FBS and 1% antibiotic–antimycotic to a concentration of 5.0 × 10^5^ cells/mL, and subjected to the HHP treatment. After treatment, cell morphology, cell viability, and cell proliferation were evaluated.

### 2.2. High Hydrostatic Pressure Application to NB1RGB Cell Suspension

**Figure 1:**
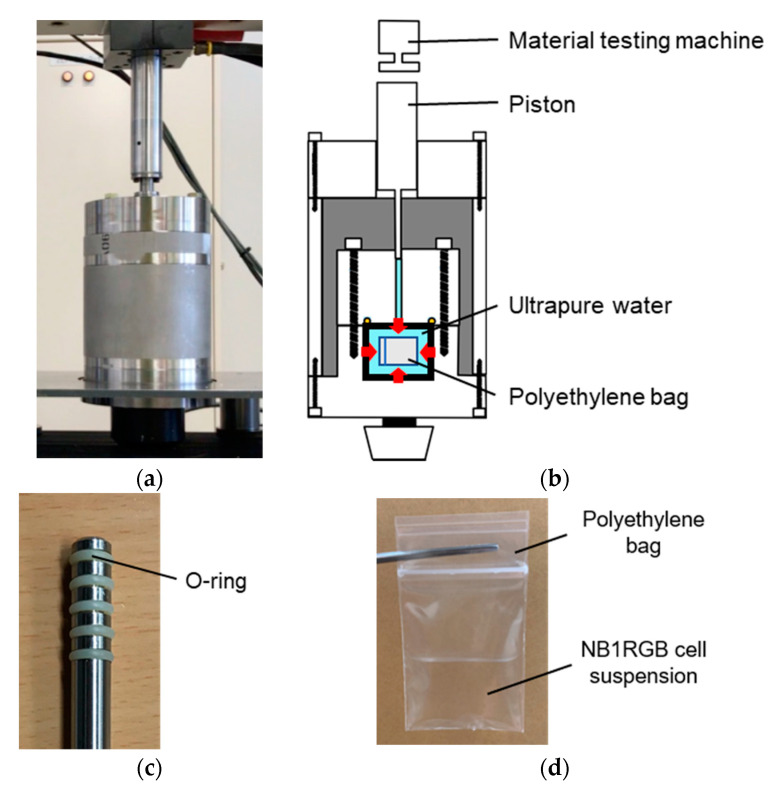

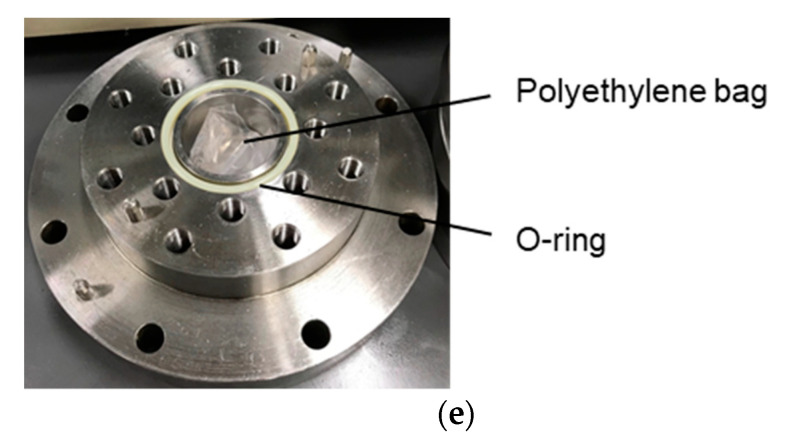
Device for applying High hydrostatic pressure (HHP) to NB1RGB cell suspension. (**a**) Overview of the device. (**b**) Cross-sectional view of the device. (**c**) Piston with five O-rings for applying HHP. (**d**) Polyethylene bag containing NB1RGB cell suspension. (**e**) Lower part of the device.

In this study, a device for applying cyclic HHP to a cell suspension was developed (Figure 1). This device consists of a piston direct pressurization vessel (Figure 1a) compressed by a material testing machine (MTS bionix858, MTS, Eden Prairie, MN, USA). HHP was applied to the cell suspension in the vessel by inserting a piston into the pressure vessel (Figure 1b). Five O-rings were attached to the side of the piston to maintain the tightness of the high-pressure container during the cyclic application of HHP (Figure 1c). The diameter and length of the piston were 8 and 105 mm, respectively, to reach a maximum HHP of 250 MPa.

A volume of 4 mL of NB1RGB cell suspension was injected into a sterilized polyethylene bag (Figure 1d) and placed at the bottom of the high-pressure vessel (Figure 1e). The high-pressure vessel was assembled with a piston placed on the upper part, and HHP was applied by pushing the piston with a material testing machine.

**Figure 2:**
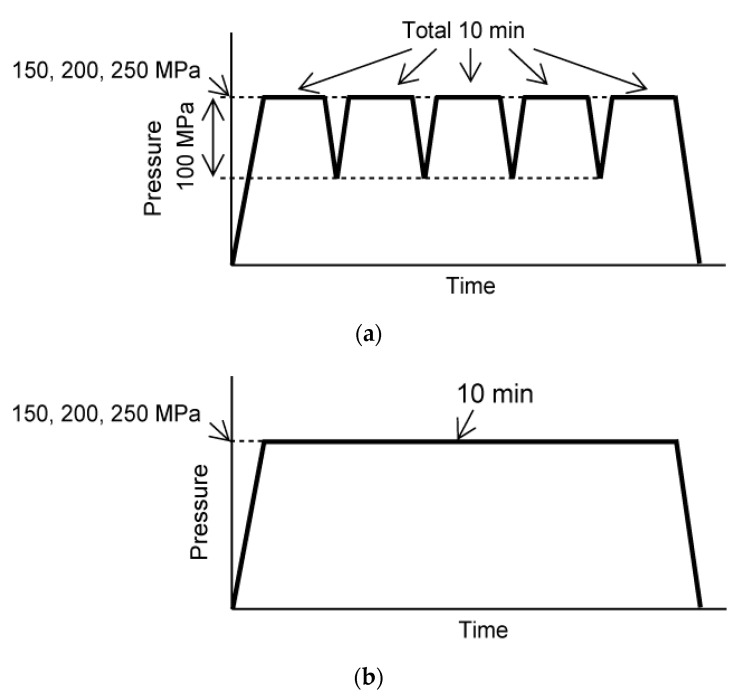
HHP displacement diagram applied to (**a**) cyclic and (**b**) static groups.

To suppress O-ring damage by HHP, the piston pressure was increased at a rate of 2.68 Mpa/s from 0 to 150 Mpa, 1.68 Mpa/s from 150 to 200 Mpa, and 0.34 Mpa/s from 200 to 250 Mpa during pressurization (Figure 2). For cyclic HHP application, the pressure was increased to 150, 200, or 250 Mpa and held for 2 min. Then, the pressure was decreased to 100 Mpa and immediately increased to the original pressure. This operation was performed five times (Figure 2a). To examine the effect of the cyclic application of HHP, static HHP was imposed on the cell suspension for the same pressurization time. For the static HHP application, HHP was continuously applied for 10 min (Figure 2b). The HHP was applied by setting the maximum pressure to 150, 200, and 250 Mpa (for a total of six experimental groups) (Table 1). For the control group, a specimen without HHP treatment was also prepared.

### 2.3. Biochemical Characterization

**Figure 3:**
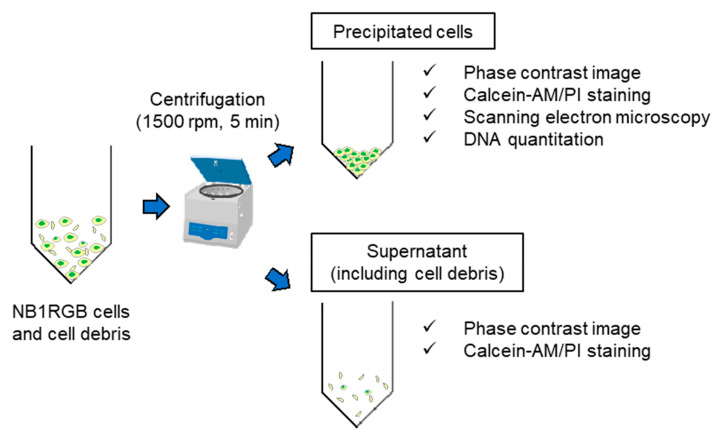
Procedure after applying HHP. After HHP was applied, NB1RGB cell suspension was transferred from the bag to the tube, then centrifuged to separate the cells and cell debris. The precipitated cells and the cells or cell debris contained in the supernatant were evaluated, respectively.

It is considered that NB1RGB cell suspension subjected to the HHP would contain cells that maintain their morphology, cells with damage to their cell membrane or nucleus, and cell debris derived from fragmented cells. In this study, the NB1RGB cell suspension was centrifuged after applying HHP and divided into centrifugal sediment (with cells and without cell debris) and supernatants (with cell debris). The viability and morphology of cells in both the centrifugal sediment and supernatant were evaluated (Figure 3).

Cell morphology and viability were evaluated by phase contrast microscopy and fluorescence microscopy. The cell membranes were observed by environmental scanning electron microscopy (E-SEM). The total DNA content of the cell suspension was also measured to evaluate the number of precipitated cells. Furthermore, the precipitated cells were seeded on a 6-well plate and cultured for 24 h to evaluate cell proliferation. As for the supernatant, the cell morphology and viability were also evaluated by phase-contrast microscopy and fluorescence microscopy.

NB1RGB cells were stained with calcein-AM and PI to evaluate cell viability and damage to the cell membrane. Calcein-AM stains the cytoplasm of living cells, whereas PI stains the cell nucleus of dead cells. PI cannot penetrate the cell membrane of living cells; therefore, it stains only the cell nucleus of dead cells. Using this nature, the damage to the cell membrane could be evaluated by PI staining. For calcein-AM/PI staining, the NB1RGB cells were first washed with phosphate-buffered saline (PBS), and then incubated with 0.1 mg/mL calcein-AM and PI in PBS for 10 min. The cells were also stained with Hoechst 33342 to evaluate the destruction of cell nuclei. The cells were incubated with 2.0 mg/mL Hoechst 33342 in PBS for 10 min. After calcein-AM/PI and Hoechst 33342 staining, the cells were observed under a fluorescent microscope (BZ-X800, Keyence, Osaka, Japan).

To observe the microstructure of the cell membrane with scanning electron microscopy (SEM), precipitated cells were fixed in 4% paraformaldehyde-phosphate buffer solution (Nacalai Tesque, Kyoto, Japan) for 30 min. Following histological fixation, the fixed cells were dehydrated to treat with 25, 50, 75, 90, and 99.5% ethanol. After dehydration, the dehydrated cells were observed using an E-SEM with an accelerating voltage of 5.0 kV (Inspect S50, FEI, Tokyo, Japan).

DNA quantification was performed for the precipitated cells immediately after HHP treatment and the cells were cultured for 24 h after HHP treatment. It has been reported that the total DNA is related to the cell number in hydrogels or living tissues [35,36]. The total DNA amount in the samples was determined using a fluorescence spectrophotometer (Qubit 2.0 Fluorometer, Life Technologies, Carlsbad, CA, USA).

### 2.4. Statistical Analysis

Most of the data are representative of three individual experiments with similar results. For each group, six samples (n = 6) were analyzed, and each data point represents the mean and standard deviation. The statistical significance of the experimental data was evaluated using the Student’s *t*-test method. *p* < 0.05 was considered statistically significant.

## 3. Results

### 3.1. Effect of Cyclic Application of HHP on Cell Morphology

**Figure 4:**
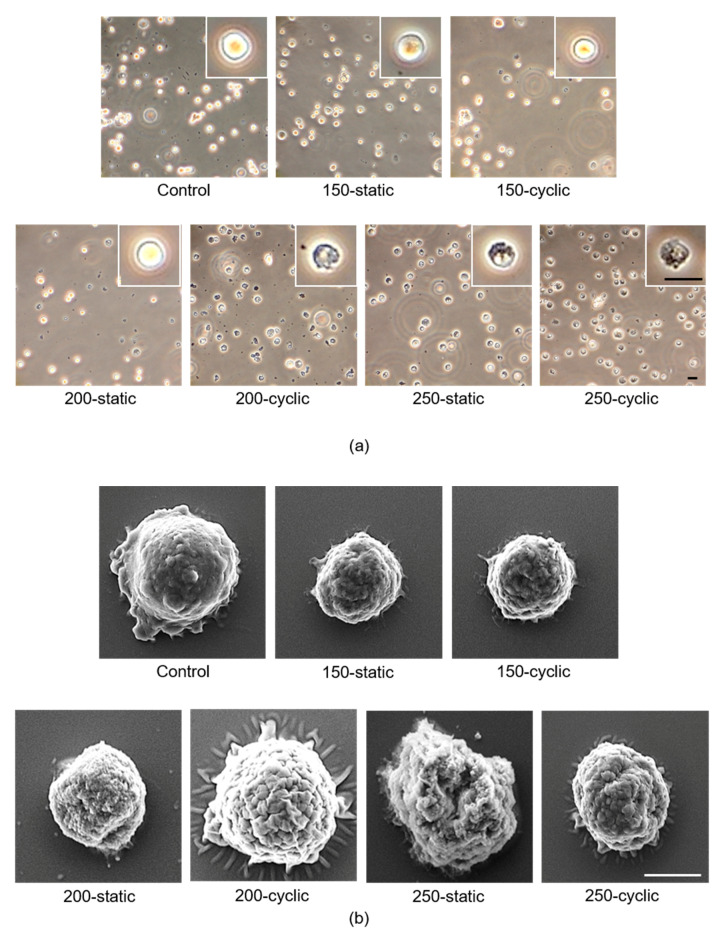

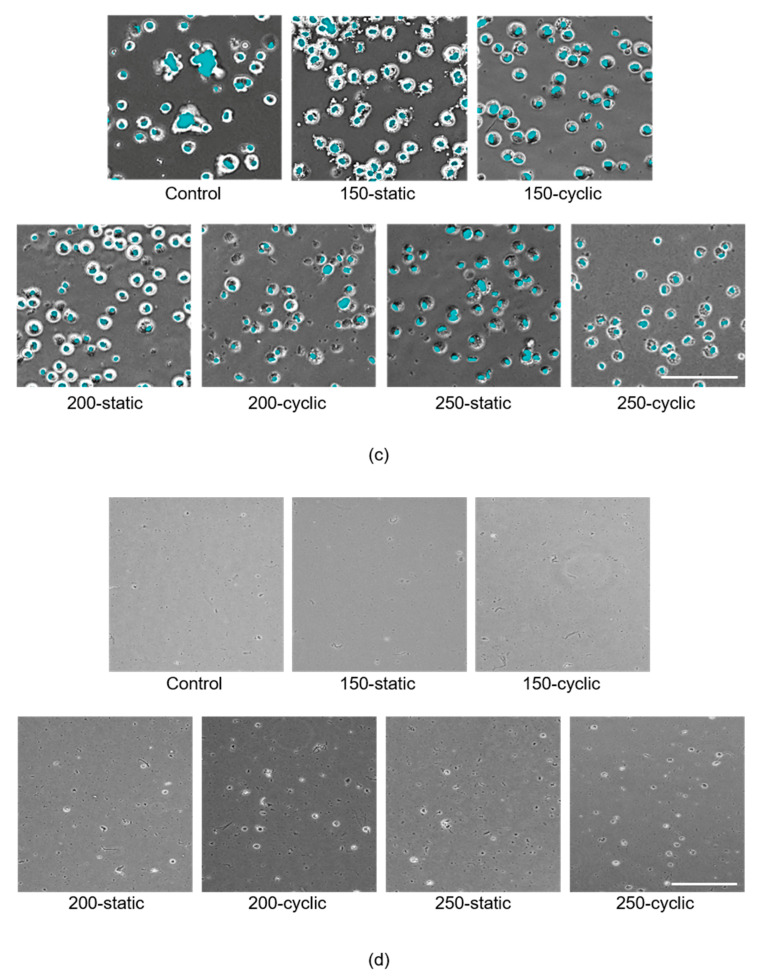
Evaluation of damage to cell membrane and nucleus of NB1RGB cells. (**a**) Phase-contrast images of NB1RGB cells precipitated by centrifugation. Scale bar: 200 μm. (**b**) Scanning electron microscopy images precipitated by centrifugation. Scale bar: 5 μm. (**c**) Hoechst staining precipitated by centrifugation. Scale bar: 100 μm. (**d**) Phase-contrast images of the supernatant. Scale bar: 300 μm.

The cell morphology in the 150-static, 150-cyclic, and 200-static groups showed a round shape, which is similar to that of the control group (Figure 4a). In contrast, the ratio of cells with a round shape decreased with HHP over 200 MPa. In the 150-cyclic and 200-static groups, more cells with dark cell regions were observed as compared to the control group. Moreover, no cells showed round shapes in the 200-cyclic, 250-static, and 250-cyclic groups, and most cells were in the dark cell region.

SEM observations were performed to evaluate the cell microstructure subjected to HHP (Figure 4b). The cell membranes subjected to static HHP of 150, 200, and 250 MPa and cyclic HHP of 150 MP showed smooth surfaces, similar to those of the control group, whereas those subjected to cyclic HHP of 200 and 250 MPa showed uneven cell membranes. Moreover, cells subjected to higher HHP showed a collapsed morphology.

The nuclei of cells were also stained with Hoechst 33342, and the green-stained area of the fluoresced image was merged with the phase-contrast image taken in the same region (Figure 4c). The nuclei of all cells were positively stained in the phase-contrast images; therefore, it was found that the cell nuclei remained in the precipitated cells in all groups.

To evaluate the cells or cell debris contained in the supernatant, the supernatant was observed using a phase-contrast microscope. Almost no cells or cell debris were observed in the control, 150-static, and 150-cyclic groups (Figure 4d). On the contrary, some cells and cell debris with fragments of cell membranes were observed at HHP of 200 and 250 MPa under both static and cyclic conditions.

### 3.2. Effect of Cyclic Application of HHP on Cell Viability

**Figure 5:**
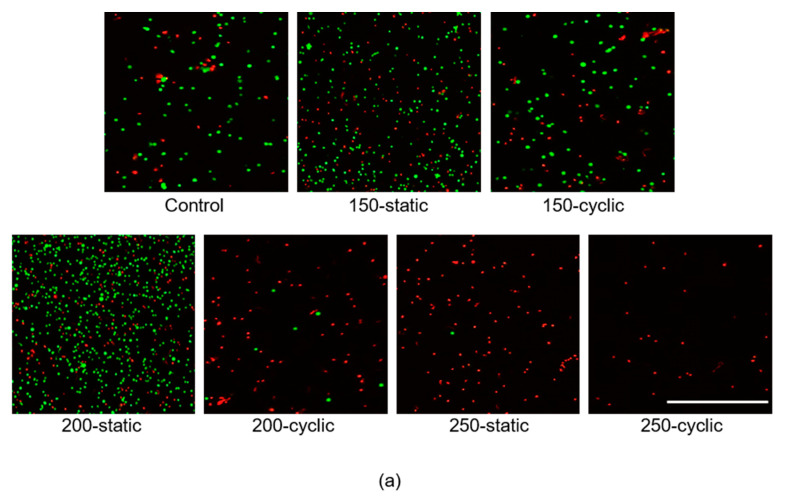

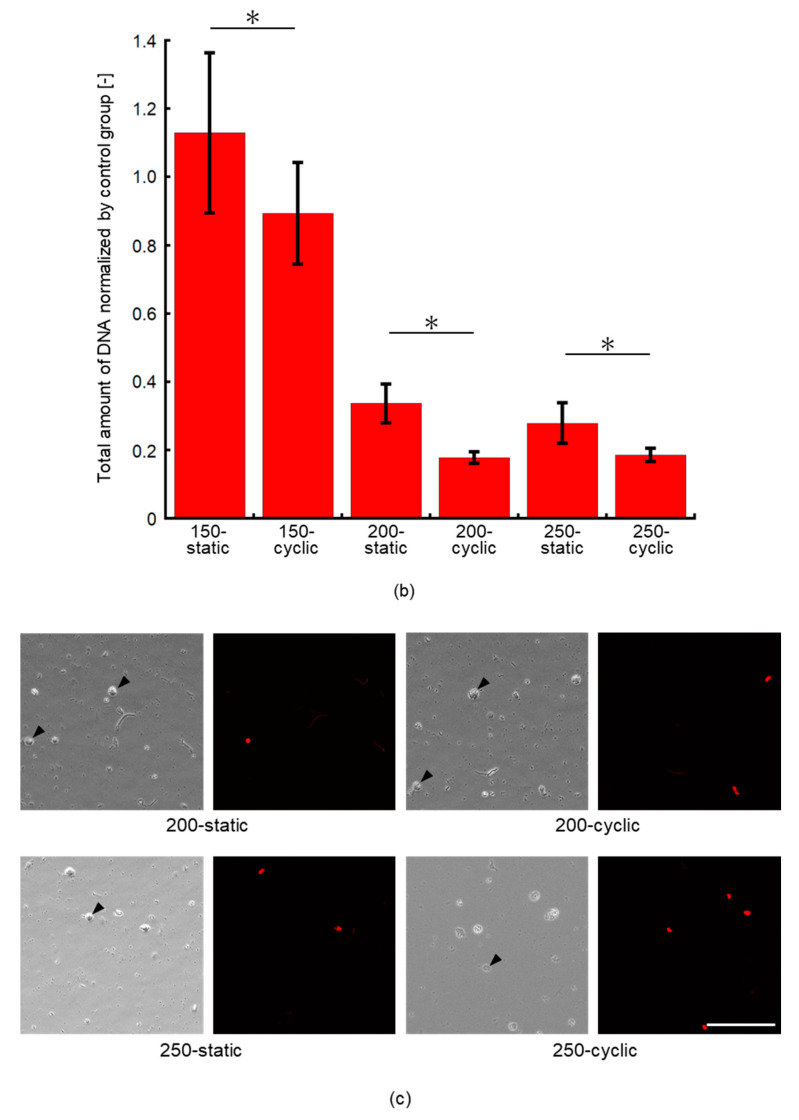
Evaluation of cell viability after HHP application. (**a**) Calcein-AM/PI staining precipitated by centrifugation. Scale bar: 500 μm. (**b**) Quantity of DNA normalized by control group in precipitated cells at 0 h. Data are presented as mean ± S.D., n = 6. * indicates significant differences between static and cyclic groups at the same pressure (*: *p* < 0.05). (**c**) Phase-contrast images (left) and images of calcein-AM/PI staining (right) at the same area in 200-static, 200-cyclic, 250-static, and 250-cyclic groups in the supernatant. Scale bar: 100 μm.

Calcein-AM and PI staining revealed that live cells were observed after applying HHP of 150 MPa, but the number of dead cells in the 150-cyclic group was larger than that in the 150-static group (Figure 5a). In the 200-static group, the proportions of live and dead cells were almost the same as those in the 150-cyclic group, whereas the proportion of dead cells in the 200-cyclic group increased with respect to that in the 150-cyclic group. Moreover, almost no live cells were observed in both the 250-static and 250-cyclic groups.

As compared to the control group, the DNA amount of cells subjected to the HHP of 200 and 250 MPa decreased to 15–35% of that in the control group (Figure 5b). There were significant differences between the static and cyclic groups at the same HHP. In addition, the difference between the DNA amount of cells subjected to static and cyclic HHP increased with an increase in the HHP level. The DNA amount in the 200-cyclic group was approximately 53% of that in the 200-static group. Moreover, that in the 250-cyclic group was approximately 67% of that in the 250-static group. The DNA amount in the cell suspensions subjected to 200 and 250 MPa of cyclic HHP application decreased by 82% and 81%, respectively, of that in the control group.

From the results of calcein-AM and PI staining, no cells were positively stained with calcein-AM in the supernatant of the experimental groups, whereas the cells stained with PI were observed (Figure 5c). This result indicates that the cells observed in the supernatant did not contain cytoplasm, which positively stained with calcein-AM. There were also some cells that were not stained with PI among the cells observed in the phase-contrast microscopic images, as indicated by the black arrows in Figure 5c. It was revealed that the nuclei of some cells were removed.

### 3.3. Effect of Cyclic Application of HHP on Cell Proliferation

**Figure 6:**
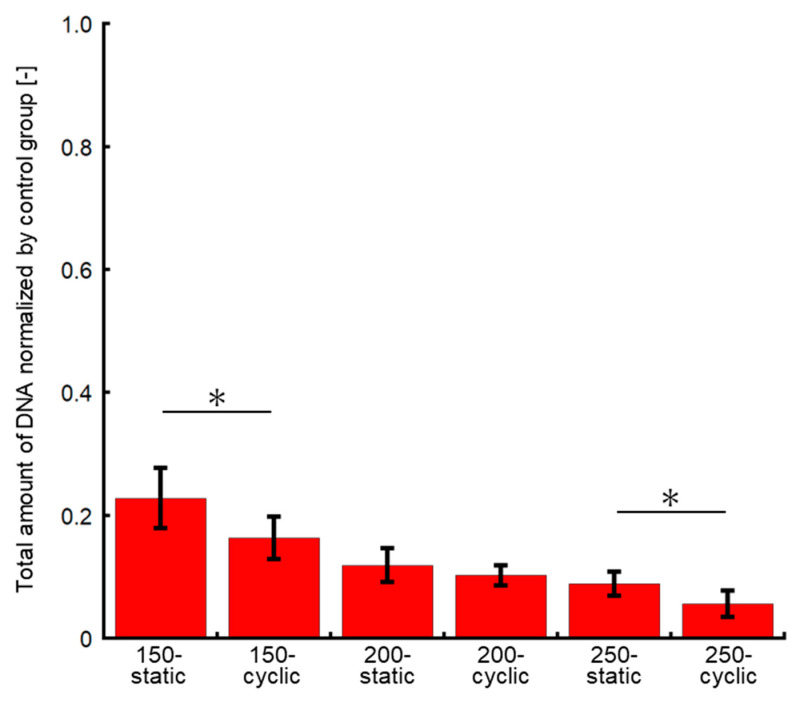
Quantity of DNA normalized by control group in precipitated cells at 24 h. Data are presented as mean ± S.D., n = 5. * indicates significant differences between static and cyclic groups at the same pressure (*: *p* < 0.05).

To evaluate the effect of HHP application on the proliferation of cells, the total DNA amount of cells cultured for 24 h after HHP application was quantified. As a result, the DNA content of cells in all groups was under 30% of that in the control group (Figure 6). The DNA content decreased as the HHP increased. Moreover, the DNA content in the cyclic HHP groups tended to show lower values as compared to those in the static HHP groups.

## 4. Discussion

In this study, the effect of cyclic application of HHP on NB1RGB cell suspension was examined to assess the hypothesis that the cell membrane would be weakened and the inflow and outflow of intracellular fluid would be promoted by cyclic application of HHP. The NB1RGB cell suspension was centrifuged after applying HHP, and both the precipitated cells and the cells contained in the supernatant were evaluated. In the precipitated cells, there were no cells in which the cell nucleus was destroyed, whereas some cells were denuclearized in the supernatant. It is considered that when the cell membrane was largely destroyed, the cytoplasm leaked, resulting in an equal cell-specific gravity to that of the surrounding medium, and the cell remained in the supernatant without precipitation, even after centrifugation.

In the NB1RGB cells precipitated by centrifugation, more cells with dark cell regions were observed as compared to the control group in the 200-cyclic, 250-static, and 250-cyclic groups. Normal cells have a three-dimensional morphology, and thus form contrast when observed with a phase-contrast microscope. On the contrary, if the cell membrane was damaged and the cell structure collapsed, the cells did not form contrast. Therefore, it is considered that HHP damaged part of the cell membrane and collapsed the cell structure in the 200-cyclic, 250-static, and 250-cyclic groups.

From the SEM observations, it is clear that degeneration of the cell membrane occurred under HHP application. These results suggested that the higher the maximum pressure, the more degenerated the cell membrane, and that the membrane in the cyclic group was more likely to degenerate than that in the static group under the same pressure. This is because the number of unclosable holes in the cell membrane did not increase in the 200-static group, but did increase in the 200-cyclic group. These results indicated that irreversible through-holes could be generated by the application of cyclic HHP.

In addition, almost no cells were positively stained with calcein-AM in the 200-cyclic, 250-static, and 250-cyclic groups. Calcein-AM could only pass through the live cell membrane and stain the cytoplasm; therefore, it was speculated that the cell membranes subjected to HHP in the 200-cyclic, 250-static, and 250-cyclic groups were partially collapsed.

At an HHP of 150 MPa, the amount of DNA in the NB1RGB cells precipitated by centrifugation was almost the same as that of the control group, whereas the amount of DNA of cells cultured for 24 h decreased to approximately 20% of that of the control group. It has been reported that mitochondria are deactivated to induce apoptosis when HHP is applied to cells [37]. At a hydrostatic pressure of 150 MPa, the cell structure showed no macroscale changes. These results suggested that the mitochondria in NB1RGB cells were deactivated to cause lower cell adhesion on the cell culture substrate.

These experimental results suggested that cyclic HHP application promoted the disruption and destruction of the cell membrane compared to the static application of HHP. Furthermore, the results suggested that cyclic HHP application induces the destruction of the cell nucleus. From these results, it was considered that the cyclic HHP application can also be employed to manufacture decellularized tissue more effectively than static application at the same magnitude of HHP. Furthermore, it is expected that the cyclic application of HHP can suppress ECM degeneration in living tissues.

## 5. Conclusions

In this study, the effect of cyclic application of HHP on the microstructure of living cells (cell membrane and nucleus) was evaluated. Our experimental results suggested that the cyclic application of HHP promoted the denaturation and collapse of the cell membrane as compared to the static application of HHP. Furthermore, it was suggested that the cyclic application induces the destruction of the cell nucleus. It is expected that cyclic HHP application can be employed to manufacture decellularized tissue more effectively than static HHP application at the same magnitude of HHP.

## Figures and Tables

**Table 1:** HHP application conditions in this study.

	Maximum Pressure (MPa)	Application Condition
150-static	150	static
200-static	200	static
250-static	250	static
150-cyclic	150	cyclic
200-cyclic	200	cyclic
250-cyclic	250	cyclic
